# The near-complete mitogenome of the critically endangered *Pseudocleopatra dartevellei* (Caenogastropoda: Paludomidae) from the Congo River assembled from historical museum material

**DOI:** 10.1080/23802359.2019.1669081

**Published:** 2019-09-25

**Authors:** Björn Stelbrink, Christian Kehlmaier, Thomas Wilke, Christian Albrecht

**Affiliations:** a Department of Animal Ecology and Systematics, Justus Liebig University Giessen, Giessen, Germany;; b Museum of Zoology, Senckenberg Dresden, Dresden, Germany

**Keywords:** Africa, Democratic Republic of the Congo, freshwater gastropods, ancient DNA, museum genomics

## Abstract

Here, we present the first near-complete mitogenome of a member of the freshwater gastropod family Paludomidae, *Pseudocleopatra dartevellei*. This Congo River species is of particular importance because the sister to the Lake Tanganyika radiation is supposed to be a paludomid riverine species. We used ancient DNA (aDNA) techniques including single-stranded DNA library preparation in order to assemble the mitogenome from historical museum material collected in 1937. The mitogenome was 15,368 bp long and showed typical characteristics as identified in other freshwater gastropods. The present phylogeny shows a closer relationship between *Pseudocleoptra dartevellei* and another non-Tanganyikan species, *Cleopatra johnstoni*.

Lake Tanganyika is home to a famous radiation of freshwater gastropods of the family Paludomidae (17 genera; Brown 1994; Neiber and Glaubrecht 2019). There is an ongoing discussion on whether Lake Tanganyika may have acted as a refuge for ancient lineages that existed in pre-lake systems such as the Congo River (Wilson et al. 2004). However, studies aiming at uncovering the biogeographical origin of the species flock have been hampered by the inaccessibility of key areas in the Congo Basin due to civil wars and unrest (Glaubrecht and Strong 2007). Under such circumstances, material conserved in institutional collections such as museums proves very valuable. This is also the case for the critically endangered riverine genus *Pseudocleopatra* (see Jørgensen 2010), which occurs in western Africa and parts of the Congo basin and which represents one of the potential sister groups of the Lake Tanganyika gastropod radiation.

A specimen of *Pseudocleopatra dartevellei* Mandahl-Barth, 1973 was collected by Edmond Dartevelle in the Congo River near Matadi, Democratic Republic of the Congo (formerly Belgian Congo; 5.8167°S 13.4667°E) in 1937 and is deposited in the Royal Museum of Central Africa, Tervuren, Belgium (RMCA 233464). The material was analyzed applying NGS protocols developed for ancient and heavily degraded DNA in the cleanroom facility of the Senckenberg Natural History Collections, Dresden.

Genomic DNA was extracted from c. 3 mm^3^ of soft tissue using the GEN-IAL All-tissue DNA-Kit (GEN-IAL GmbH, Troisdorf, Germany) protocol for forensic material and converted into a single-indexed single-stranded Illumina sequencing library (Gansauge and Meyer 2013; Korlević et al. 2015), including an uracil-DNA glycosylase (UDG) treatment. Shotgun sequencing was conducted on an Illumina MiSeq^®^ platform (75 bp paired-end reads). After adapter trimming, quality filtering, and duplicate removal, reads mapping to a set of non-mollusk mitogenomes were excluded using FastQ Screen 0.11.4 (Wingett et al. 2018). The reduced readpool (c. 13.4 million reads) was then mapped against *Cerithidea obtusa* (NC_039951) using BWA 0.7.15 (Li and Durbin 2009). The obtained fragmentary consensus was used as a seed reference for a two-step baiting and iterative mapping approach in MITObim 1.9 (Hahn et al. 2013), with an allowed mismatch value of 2, resulting in a single high-quality contig (assembled reads: 24,308; average read length: 51 bp; maximum coverage = 211; average coverage = 82). The contig was visualized and checked for coverage and assembly artifacts in Tablet 1.16.09.06 (Milne et al. 2013), and the authenticity of the mapped reads was tested with mapDamage 2.0 (Jónsson et al. 2013). The contig was finally annotated on the MITOS2 Web Server (Bernt et al. 2013; reference dataset = RefSeq 63 Metazoa).

The almost complete, annotated mitogenome has 15,368 bp (with only three ambiguous sites) and is deposited at GenBank (MN082637). The base composition was biased towards a high A + T content (63.72%; A = 28.16%, T = 35.56%), whereas the G + C content was comparatively low (36.26%; G = 19.44%, C = 16.82%). The majority of protein-coding genes (PCGs) started with an ATG (i.e. AUG) codon and stopped with a TAA codon. Gene arrangement on the light strand was: *tRNA^Cys^*, *tRNA^Arg^*, *tRNA^Ala^*, *tRNA^Asn^*, *tRNA^Trp^*, *tRNA^Glu^*, *tRNA^Tyr^*, *tRNA^Lys^*, *COX3*, *tRNA^Met^*, *COB*, *ND6*, *tRNA^Pro^*, *ND1*, *tRNA^Leu2^*, *tRNA^Leu1^*, 16S-*rRNA*, *tRNA^Val^*, *tRNA^Gly^*, *tRNA^Thr^*, and *12S-rRNA*. On the heavy strand, gene order was: *tRNA^Ser1^*, *ND2*, *tRNA^Asp^*, *ATP8*, *ATP6*, *tRNA^Ile^*, *ND3*, *COX1*, *COX2*, *tRNA^Ser2^*, *tRNA^Gln^*, *ND4L*, *ND4*, *tRNA^His^*, *ND5*, and *tRNA^Phe^*. Overlaps were identified between *COB* and *ND6* (47 bp), *tRNA^Thr^* and *12S-rRNA* (3 bp), *ND2* and *tRNA^Asp^* (2 bp), and *ND5* and *tRNA^Phe^* (1 bp).

For a phylogenetic reconstruction of the Paludomidae, available mitochondrial DNA sequences were downloaded from GenBank that refers to the study of Wilson et al. (2004). A maximum-likelihood tree based on partial sequences of *COX1* and 16S-*rRNA* was constructed (Figure 1) using RAXML-HPC BLACKBOX 8.2.10 (Stamatakis 2014; settings: GTR + Γ model, codon partition scheme) on the CIPRES Science Gateway (Miller et al. 2010). Accordingly, *Pseudocleopatra dartevellei* is sister to *Cleopatra johnstoni* from Lake Mweru, a lake in the southeastern corner of the Congo drainage system. Although intergeneric relationships are often poorly supported, the present phylogeny supports a previous study that suggested a non-monophyly of the Lake Tanganyika paludomids (see Wilson et al. 2004).

**Figure 1.**
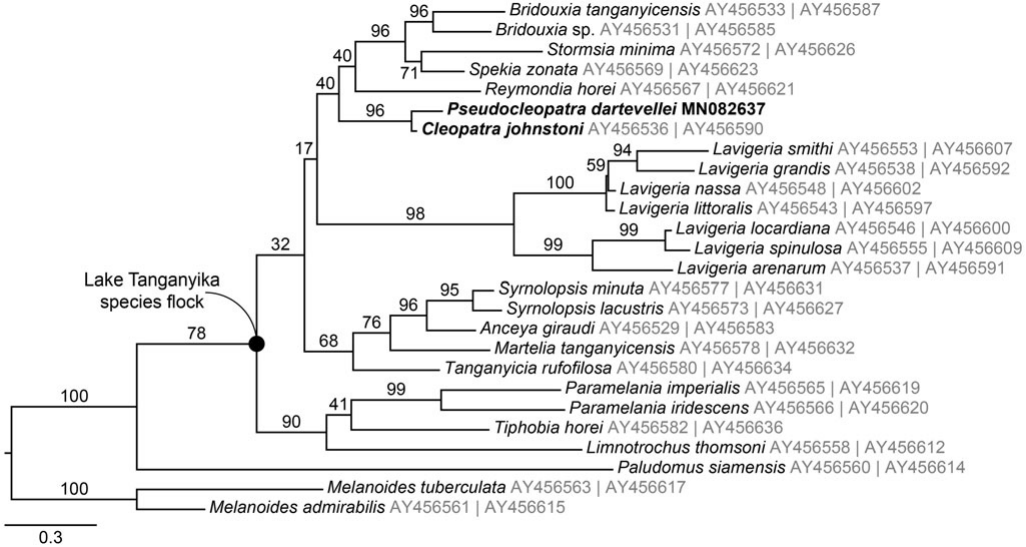
*COX1*–16S-*rRNA* maximum-likelihood phylogeny based on GenBank data (Wilson et al. 2004). The non-Tanganyikan species *Pseudocleopatra dartevellei* (Congo River) and *Cleopatra johnstoni* (Lake Mweru, Zambia) are marked in bold; the two *Melanoides* species were used as outgroups. GenBank accession numbers are shown in grey (left: *COX1*, right: 16S-*rRNA*).
